# Study on Flocculation Behavior of Cr(VI) Using a Novel Chitosan Functionalized with Thiol Groups

**DOI:** 10.3390/polym15051117

**Published:** 2023-02-23

**Authors:** Yuelong Zhao, Peng Zhang, Wei Zhang, Yali Fan

**Affiliations:** 1 College of Environmental Science and Engineering, Taiyuan University of Technology, Taiyuan 411201, China; 2 Upgrading Office of Modern College of Humanities and Sciences of Shanxi Normal University, Linfen 041000, China; 3 College of Civil Engineering, Hunan University of Science and Technology, Xiangtan 411201, China; 4 College of Hydraulic Engineering, Changsha University of Science & Technology, Changsha 410114, China

**Keywords:** hexavalent chromium, chitosan, reduced glutathione, chelating agent, amidation reaction, flocculation

## Abstract

In this study, CTS-GSH was prepared by grafting thiol (–SH) groups onto chitosan (CTS), which was characterized through Fourier Transform Infrared (FT-IR) spectra, Scanning Electron Microscopy (SEM) and Differential Thermal Analysis–Thermogravimetric Analysis (DTA-TG). The performance of CTS-GSH was evaluated by measuring Cr(VI) removal efficiency. The –SH group was successfully grafted onto CTS, forming a chemical composite, CTS-GSH, with a rough, porous and spatial network surface. All of the molecules tested in this study were efficient at removing Cr(VI) from the solution. The more CTS-GSH added, the more Cr(VI) removed. When a suitable dosage of CTS-GSH was added, Cr(VI) was almost completely removed. The acidic environment at pH 5–6 was beneficial for the removal of Cr(VI), and at pH 6, the maximum removal efficiency was achieved. Further experimentation showed that with 100.0 mg/L CTS-GSH for the disposal of 5.0 mg/L Cr(VI) solution, the removal rate of Cr(VI) reached 99.3% with a slow stirring time of 8.0 min and sedimentation time of 3 h; the presence of four common ions, including Mg^2+^, Ca^2+^, SO_4_^2−^ and CO_3_^2−^, had an inhibitory effect on CTS-GSH’s ability to remove Cr(VI) from the aqueous solution, and more CTS-GSH was needed to reduce this inhibiting action. Overall, CTS-GSH exhibited good results in Cr(VI) removal, and thus has good potential for the further treatment of heavy metal wastewater.

## 1. Introduction

It is well known that hexavalent chromium (Cr(VI)) is a carcinogen [1,2,3]. At present, a large amount of Cr(VI)-containing wastewater is discharged into natural bodies of water mainly resulting from the electroplating, tanning, chemical, pigment, metallurgy industrial processes [4]. Cr(VI) is often found in aquatic environments [5,6,7], and it is therefore important for us to control, remediate and prevent its further release into the environment. Coagulation–flocculation has received extensive attention in wastewater treatment because it is cost-effective and easy to operate [8,9,10]. However, traditional coagulants are not able to achieve reliable results in Cr(VI) removal, and there are even some drawbacks, such as secondary pollution and relatively high costs [11,12]. Therefore, developing new and eco-friendly coagulants to control Cr(VI) pollution in water is critical.

Recently, using emerging natural polymers such as lignin [13], algal alginate [14] and starch [15] to develop a series of polymeric flocculants has received significant attention. Chitosan (CTS), a biological polysaccharide, is a natural cationic polymer that is abundant in amino groups (–NH_2_), hydroxyl groups (–OH) and carboxyl groups (–COOH) [16,17]. Cr(VI) often exists as oxyanions (e.g., Cr_2_O_7_^2−^ or CrO_4_^2−^) in aqueous environments, and CTS carries a positive charge; thus, an interaction is able to occur between CTS chains and negatively charged oxyanions [16]. Additionally, its fascinating features, such as low toxicity, biocompatibility, biodegradability, renewability and good chelating ability, make it an important raw material for the development of coagulants [18,19]. While CTS has been widely used, it is generally not used to treat heavy-metal-polluted wastewater (e.g., Cr(VI)) as it has a small number of metal binding sites [20]. Therefore, there are many researchers devoted to its chemical modification to improve the performance of CTS. For instance, Martínez-Quiroz et al. [20] used carbamoyl benzoic acids as a modifier to enhance the metal-binding abilities of a chitosan matrix. In addition, Huang et al. [16] introduced a strong cationic 3-chloro-2-hydroxypropyl trimethyl ammonium to CTS chains, which could neutralize the negative charge of colloids through adsorption and charge neutralization. Combining surface functionalization with soft acid and alkali theory, especially via incorporating sulfur-containing functional groups (e.g., thiol, xanthate) grafted onto coagulants, showed it can effectively chelate with metal ions and form stable chelate compounds [21,22,23]; the chelate is then separated by flocculation and precipitation to achieve the purpose of removing metal ions from the water. Many new composite materials have been developed by grafting thiol or xanthate onto various organic compounds to modify the physical and chemical properties, so that these modified composite materials have an excellent ability to remove various metal ions [24,25].

Glutathione (GSH) is a kind of biological non-protein mercaptan. At high concentrations [26,27], it plays an important role in maintaining the biological function of cells; as a tripeptide compound consisting of three amino acids, glutamic acid, cysteine and glycine, it has many important physiological functions, such as detoxification, antioxidation, liver protection, cell homeostasis and immunity enhancement [28]. As an antioxidant, it can not only protect important proteins from oxidation in the human body, but also remove free radicals produced in the process of metabolism. Therefore, GSH has been prepared in the form of an orally ingested liquid as a health product [29]. GSH molecules possess multiple coordination groups, including carboxyl, thiol (-SH) and amino groups. Moreover, heavy metal ions have a strong affinity for S elements, and thiol is a very important functional group in GSH’s molecular structure, having the ability to combine with metal ions to form non-toxic or low-toxicity complexes [30,31,32]. As an alternative, flocculation is an environmentally friendly, simple, stable, safe and increasingly important process in pollution control [33,34,35]. Flocculation is a typical step in heavy-metal-polluted water treatment, and as the flocculant is the core of this technology, it is very important to synthesize or choose a suitable flocculant [36,37,38]. In this study, we designed a novel kind of metal-chelating coagulant by grafting GSH onto a CTS framework. A functionalized CTS (denoted as CTS-GSH) was prepared through an amide reaction to introduce GSH to CTS. Thus, the CTS-GSH contained multiple coordination groups (e.g., a carboxyl group, thiol group and amino group), which could not only combine with Cr(VI) to form low-toxicity Cr(III) but also effectively eliminate Cr(VI) from an aqueous solution.

We aimed to test if CTS-GSH could be applied to eliminate Cr(VI) from an aqueous solution. In the present work, a facile method for inducing an amide reaction to introduce functional groups (especially thiol groups) to chitosan chains was developed. The CTS-GSH was characterized by FT-IR, SEM and DTA-TG. Batch experiments were performed, and the influencing factors, including the initial concentration of Cr(VI), the CTS-GSH dosage, pH, slow stirring time, sedimentation time, organic complexing agents and inorganic coordination ions on Cr(VI), were assessed.

## 2. Materials and Methods

### 2.1. Materials

The chitosan (degree of deacetylation ≥ 90%) was a biological reagent that was obtained from Shanghai Cloud Chemical Co., Ltd. (Shanghai, China). Reduced glutathione was obtained from Shanghai Cheng Shao Biological Technology Co., Ltd. (Shanghai, China). 1-ethyl-(3-methyl two aminopropyl) carbon two imine hydrochloride (EDAC) was obtained from Shanghai Siyu Chemical Technology Co., Ltd. (Shanghai, China). N-hydroxy succinimide (NHS) was obtained from Beijing Hua MEIKO Biotechnology Co., Ltd. (Beijing, China). All other chemical reagents were analytical-grade or higher, and all solutions were prepared using ultrapure water. The PB-10 acidity meter was bought from Sartorius Scientific Instrument Co., Ltd. (Beijing, China). The ZR4-6 program-controlled jar test apparatus used for coagulation was obtained from Zhongrun Water Industry Technology Development Co., Ltd. (Shenzhen, China).

### 2.2. Preparation of CTS-GSH

**Figure 1:**
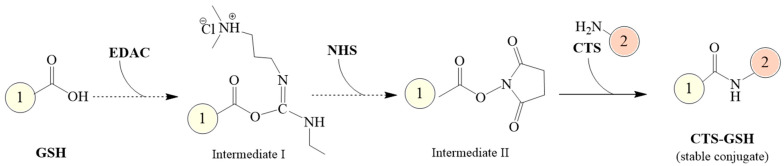
Synthetic route of CTS−GSH.

Using CTS and GSH as the main raw materials, NHS as a protective agent and EDAC as a cross-linking agent, an amidation reaction was induced to prepare a novel composite coagulant thiolated chitosan (CTS-GSH). The CTS-GSH was prepared according to Figure 1.

The steps for CTS-GSH preparation were as follows: Firstly, 0.50 g of CTS was dissolved in 5.0 mL of HCl (1.0 mol/L) solution in a beaker, and 120 mL of ultrapure water was added to dissolve it completely. Then, it was transferred to a 250 mL three-necked flask. Secondly, 0.25 g of EDAC, 0.45 g of NHS and 0.50 g of GSH were subsequently added to the abovementioned solution, using NaOH (0.10 mol/L) to adjust the solution’s pH value to 3.5. The reaction was carried out for 5.0 h under magnetic stirring, and then the non-purified product was obtained. Then, the product was purified by maintaining the volume ratio of the non-purified product to ethanol at 1:5 and oscillating the solution uniformly for 1.0 h. After oscillating, the solution was centrifuged at 3000 rpm for 10 min and filtered through a 0.45 μm filter to obtain the product. Then, the product was washed 3 times with ethanol. Finally, the product was dried in a vacuum at 60 °C for 24 h, and the light-yellow solid product (CTS-GSH) was finally obtained.

### 2.3. Flocculation Experiments and Analytic Method

**Figure 2:**
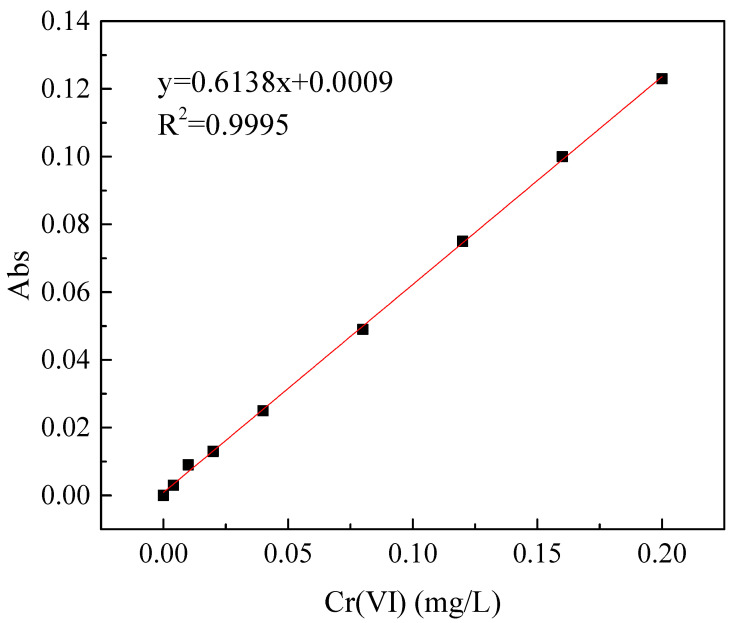
The calibration curve of Cr(VI) concentration.

A ZR 4–6 stirring machine (Shenzhen Zhongran Water Industry Technology Development Co., Ltd., Shenzhen, China) with six stirrers was used in this experiment. The flocculation stirring experiments were carried out in a beaker. The volume of the solution was 250 mL, and the concentration of Cr(VI) in the solution was 5.0 mg/L. After adding an appropriate amount of CTS-GSH, stirring was performed at 210 r/min for 1 min and at 50 r/min for 6 min. Then, after quiescent settling for 1 h, water samples from 2 cm below the surface of the liquid were collected. The Cr(VI) concentration of water samples was determined using the diphenylcarbazide colorimetric method [38]. Under acidic conditions, Cr(VI) can react with diphenylcarbazide to form a purplish-red chelate. An ultraviolet spectrophotometer (at 540 nm) was used to measure the absorbance value of water samples containing different concentrations of Cr(VI). The calibration curve of Cr(VI) concentration is shown in Figure 2.

### 2.4. Characterization Analysis of CTS-GSH

The FT-IR spectra of CTS-GSH were obtained using a Nicolet 6700 IR spectrometer (Thermo Fisher Scientific Inc., Waltham, MA, USA). The SEM pictures of CTS-GSH were taken using a JSM-6380LV scanning electron microscope (Japan Electron Optics Laboratory Co., Ltd, Tokyo, Japan). The DTA-TG image of CTS-GSH was obtained using a DTG-60H (SHIMADZU, Tokyo, Japan), and the analysis was performed with a heating rate of 10 °C/min at a temperature range of 25~600 °C.

## 3. Results and Discussion

### 3.1. FT-IR Analysis

**Figure 3:**
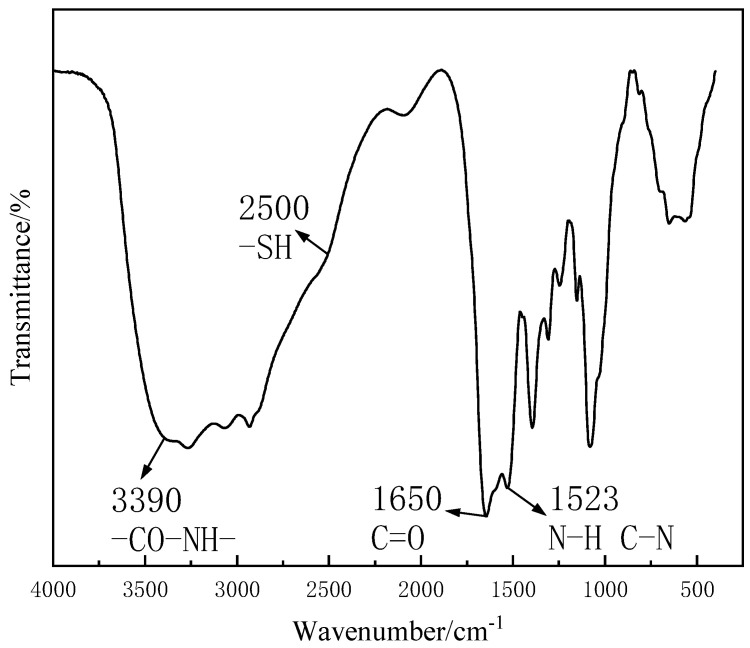
FT-IR spectrum of CTS-GSH.

As shown in Figure 3, there was a secondary amide group (-CO-NH-)-characteristic absorption peak near 3390 cm^−1^. The C-N bond of the amide compound was a double bond, and the two substituents of the N atom were larger. Besides the steric hindrance effect of the two atoms hindered by the amino space, the secondary amide was mainly observed in the presence of the S^−^ trans structure [39]. However, internal hydrogen bonds cannot be generated by trans generation, and due to the existence of intermolecular hydrogen bonding and the concentration effect, the stretching vibration of N-H in the amide bond moved towards a low wave number [40]. At 1650 cm^−1^, the stretching vibration frequency of a secondary amide, “amideⅠ”, C=O, appeared, and the characteristic absorption peak could be clearly seen. The “amide II” of the secondary amide at 1523 cm^−1^ was caused by the co-frequency of the N-H angular vibration and the C-N expansion vibration [41]. At around 2500 cm^−1^, weak thiol (-SH) stretching vibrations appeared in CTS-GSH [42,43,44], and the absorption peak was not obvious, which was due to the weak absorption intensity and the shift from intermolecular hydrogen bonding to the low wave number. It was proved that CTS and GSH had an amide reaction under the action of EDAC and NHS. The thiol group was successfully introduced into the CTS molecule.

### 3.2. SEM Analysis

**Figure 4:**
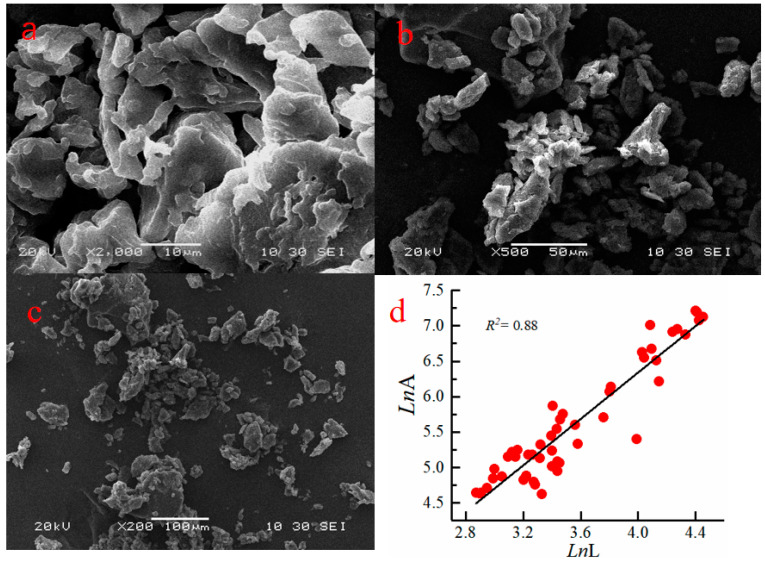
SEM pictures and the relationship between lnL and lnA of CTS-GSH. (**a**) Scanning electron microscope at 2000× magnification, (**b**) Scanning electron microscope at 500× magnification, (**c**) Scanning electron microscope at 200× magnification, (**d**) Linear correlation between LnL and LnA.

Figure 4a–c show the SEM pictures obtained to investigate the amorphous morphological of CTS-GSH. Figure 4d shows the relationship between lnL and lnA of CTS-GSH. Figure 4a,b show the space network structure of the CTS-GSH, with a size of 10µm–5µm and a rough surface with holes, which helped it adsorb pollutants in the aqueous solution, leading to compact flocs and rapid subsidence [45,46]. In order to more effectively analyze the surface structure of the CTS-GSH, particle size analysis was performed in the area indicated by the arrow in Figure 4c. The particle size was measured and analyzed using Image-Pro Plus 6.0 software [25,47]. The fractal dimension of the calculated CTS-GSH samples reflected the relationship between the perimeter and its area [48]. Additionally, its product perimeter (L) had a linear correlation with the logarithm of area (A). The correlation coefficient was 0.88, and the fractal dimension was 1.64, which is relatively small. This may be due to the large number of pores between particles, which could have helped in adsorbing pollutants [49].

### 3.3. DTA-TG Analysis

**Figure 5:**
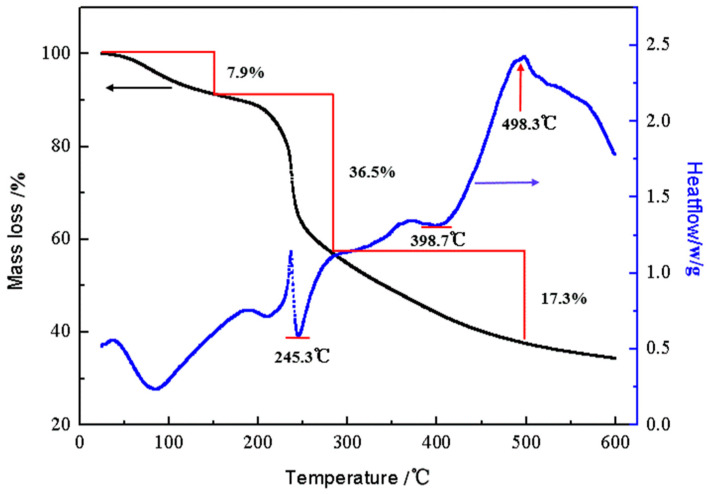
DTA-TGA images of CTS-GSH.

As shown in Figure 5, there were two obvious endothermic peaks in the differential thermal curve. The initial decomposition temperature was 50 °C, and the first endothermic peak occurred at 85.7 °C, resulting in a solid–solid phase transition. At about 200 °C, the glass transition (secondary phase transition) occurred. At the second endothermic peak temperature of 245.3 °C, the crystallization phenomenon occurred. At about 373.3 °C, the crystallized CTS-GSH gradually became exothermic and melted. When the main chain of CTS-GSH was oxidized at approximately 398.7 °C, a weak endothermic peak appeared. The temperature continued to rise, and the decomposition and gasification happened near 498.3 °C. As can be seen in Figure 5, with rising temperature, three distinct weight reduction stages were seen on the CTS-GSH thermogravimetric curve. The first stage occurred between 50 and 150 °C. The first weight loss curve appeared when CTS-GSH lost 7.9% of its weight, which may have been caused by the evaporation and sublimation of the tested water. The second stage occurred between 200 and 300 °C, with a fast and continuous weight loss curve and weight loss of 33.88%. This was probably due to the breaking of weak bonds in CTS-GSH, such as amide groups, the release of noncarbon atoms, the remaining skeleton and the release of crystal water in CTS-GSH. The last stage occurred between 300 and 500 °C. There was a slow weight loss curve, which may have been due to the gradual oxidation of the remaining carbon chain in CTS-GSH and the breaking of bonds at the beginning of the process [50].

### 3.4. Study on the Removal of Cr(VI) by CTS-GSH

#### 3.4.1. Effect of CTS-GSH Dosage

An experiment was performed to evaluate the effects of CTS-GSH dosage on Cr(VI) removal under the condition of pH 7.

**Figure 6:**
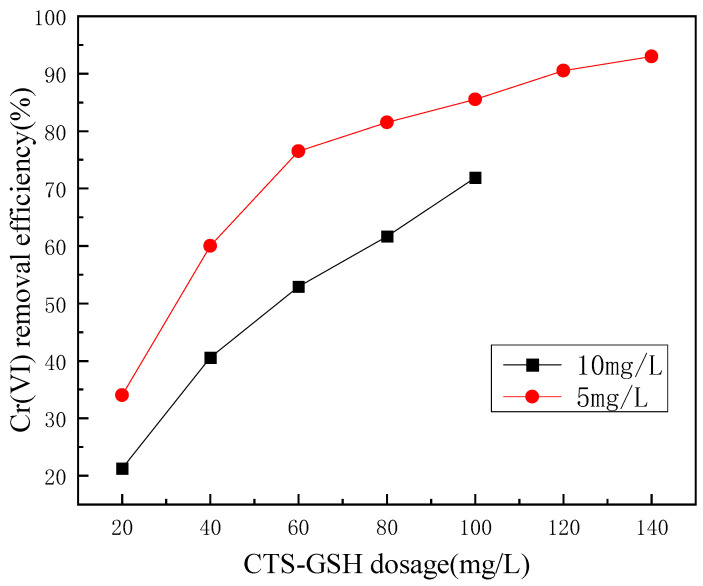
Effect of CTS-GSH dosage on Cr(VI) removal.

As shown in Figure 6, as CTS-GSH dosage increased within a certain range, the removal rate of Cr(VI) gradually increased and remained stable. The reason for the analysis was that the initial concentration of Cr(VI) was constant (5.0 mg/L and 10.0 mg/L), and the CTS-GSH contained amino, carboxyl, thiol and other electron-donating coordination groups. When the CTS-GSH dosage was insufficient, there were not enough coordination groups to interact with Cr(VI) in the solution to form the chelate complex, resulting in its poor removal. As the amount of CTS-GSH increased, the coordination group that coordinated with Cr(VI) increased, the chelate complex was formed, and the Cr(VI) removal efficiency increased accordingly. Finally, through the chelate complex’s mutual adsorption and bridging, the floc settled and separated from the water, achieving the removal of Cr(VI). The concentration of Cr(VI) in the solution was constant, so when the CTS-GSH concentration reached a certain level, the change in the Cr(VI) removal efficiency became smaller and finally tended to be stable [51].

#### 3.4.2. Effect of pH

An experiment was performed to evaluate the effects of pH on Cr(VI) removal with 80.0 mg/L CTS-GSH and an initial concentration of Cr(VI) of 5.0 mg/L.

**Figure 7:**
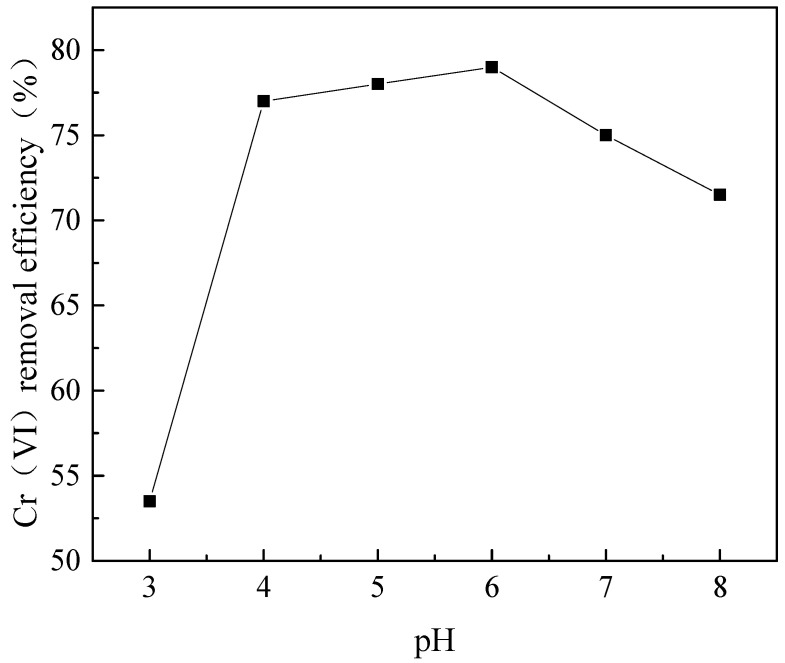
Effect of pH on Cr(VI) removal.

As shown in Figure 7, when the pH increased from 3.0 to 8.0, the Cr(VI) removal efficiency first increased and then decreased. The Cr(VI) removal efficiency with CTS-GSH changed from a low value of 53.5% when the pH value was 3.0 to a maximum of 79.0% when the pH value was 6.0. A possible reason for this is that when the pH was low, there were many H^+^ ions in the solution. Additionally, the empty orbitals of H^+^ were susceptible to accepting lone-pair electrons on nitrogen, oxygen and sulfur [52], which weakened the chelation of Cr(VI) with the coordination groups (e.g., carboxyl, thiol, amino), thereby hindering the formation of chelates, so that the Cr(VI) removal efficiency decreased. As the pH value increased, the concentration of H^+^ decreased, and the chelation effect was relatively enhanced, so the removal effect was improved. However, when the pH was higher than 6.0, the Cr(VI) removal efficiency decreased. A possible reason for this is that positively charged CTS-GSH would react with OH^−^, leading to a decrease in the level of solubility of CTS-GSH in alkaline conditions [53], which might also be the reason for the decreased removal efficiency.

#### 3.4.3. Effect of Slow Stirring Time and Sedimentation Time

An experiment was performed to evaluate the effects of different slow stirring times on Cr(VI) removal under the condition of pH 7 with 80.0 mg/L CTS-GSH when the initial concentration of Cr(VI) was 5.0 mg/L and the slow stirring rate was 50 r/min.

**Figure 8:**
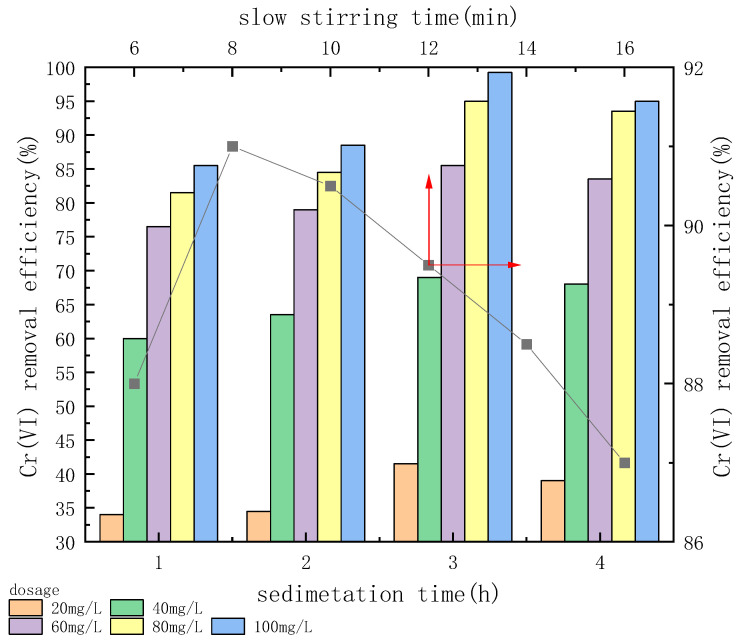
Effect of slow stirring time and sedimentation time on Cr(VI) removal.

As shown in Figure 8, when slow stirring time increased within a certain range, the Cr(VI) removal efficiency first increased and then decreased. Cr(VI) removal efficiency increased when the slow stirring time was increased from 6 min to 8 min, and when the slow stirring time was 8.0 min, it reached a maximum of 91.0%. When the slow stirring time was prolonged, the Cr(VI) removal efficiency decreased, and it was 87.0% when the slow stirring time was 16.0 min. The results show that a short slow stirring time caused the coagulant to fail after hydrolysis due to fully colliding with Cr(VI), which limited the chelation between Cr(VI) and CTS-GSH. Additionally, CTS-GSH and Cr(VI) did not collide with each other effectively; thus, the flocculation effect was relatively weak. As the slow stirring time increased, CTS-GSH and Cr(VI) could completely combine, forming small floc particles and an intermolecular interaction, thus resulting in better flocculation. However, at a long slow stirring time, the shear stress intensity may increase, thus breaking formed flocs [54]. As a result, this would again lead to the release of pollutants into the solution.

As shown in Figure 8, as the sedimentation time increased, the Cr(VI) removal efficiency first increased and then decreased. When the sedimentation time increased from 1.0 h to 3.0 h, the Cr(VI) removal efficiency increased. At a sedimentation time of 3.0 h, when the CTS-GSH dosage was 100 mg/L, the removal rate of Cr(VI) reached 99.3%. In addition, when the sedimentation time was increased to 4.0 h, the corresponding Cr(VI) removal efficiency was slightly reduced. The reason for the above phenomenon might be that when the sedimentation time was relatively short, the Cr(VI) chelated with CTS-GSH and aggregated to form small flocs [55]. Additionally, when the dosage of CTS-GSH was insufficient, good chelation and flocculation were unlikely to occur, so the removal rate of Cr(VI) was poor. When the sedimentation time increased, under the dual roles of the gravity settlement and the thermal motion of the molecules, the flocs gradually became larger and slowly settled down, and the Cr(VI) removal efficiency increased to an optimal value. However, as the sedimentation time was continuously increased, under the effect of the thermal motion of molecules, the flocs formed by CTS-GSH and Cr(VI) were re-stabilized, leading to the release of a little Cr(VI) once again into the solution [56]. In conclusion, choosing the best sedimentation time could serve to promote CTS-GSH’s function as a metal chelator.

#### 3.4.4. Effect of Organic Complexing Agent

An experiment was performed to evaluate the effects of an organic complexing agent for Cr(VI) removal under the condition of pH 7 when the initial concentration of Cr(VI) was 5.0 mg/L.

**Figure 9:**
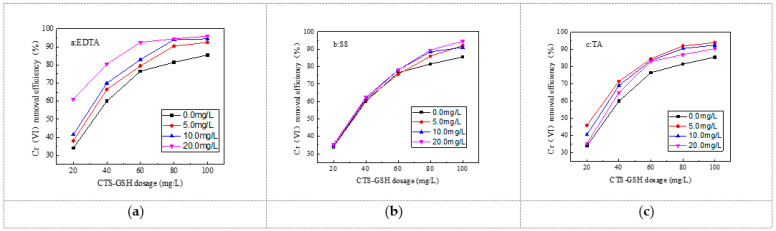
Effect of organic complexing agent on Cr(VI) removal. (**a**) Effect of EDTA on Cr(VI) removal. (**b**) Effect of SS on Cr(VI) removal. (**c**) Effect of TA on Cr(VI) removal.

It can be seen from Figure 9 that ethylenediaminetetraacetic acid disodium salt (EDTA), sodium salicylate (SS) and tartaric acid (TA) can serve to promote the Cr(VI) removal rate. From Figure 9a,b, it can be seen that the Cr(VI) removal rate increased with EDTA and SS. When the EDTA and SS concentration was 20 mg/L, with a CTS-GSH dosage of 100 mg/L, the Cr(VI) removal rate was 96.0% and 93.5%, respectively. TA had different effects depending on the concentration used; a low concentration of TA better promoted Cr(VI) removal efficiency. For example, with only 5.0 mg/L TA and 100 mg/L CTS-GSH, 94% Cr(VI) removal could be obtained. The major reason for this phenomenon is that EDTA has a strong chelating capability, and can thus form a chelate ring structure with Cr(VI) [57]. With an increase in the concentration of EDTA, more chelate structure formations appeared, which helped the coagulant to play bridging and sweeping roles. SS is rich in hydroxyl and carboxyl groups that have a strong coordination capability [58]. It can form a stable complex with Cr(VI). At an increased SS concentration, more complexes formed, which helped the coagulant in adsorption–bridging and sweeping, so the removal effect increased. TA is rich in polar groups that have a certain chemical adsorption and reduction capability [59]. At a low concentration of TA, it can react with Cr(VI) in the water to form more complexes to effectively remove Cr(VI). However, with excess TA, it consumes more of the thiol groups in CTS-GSH, potentially causing a negative effect on Cr(VI) removal.

#### 3.4.5. Effect of Cations

An experiment was performed to evaluate the effects of cations including Mg^2+^ and Ca^2+^ on Cr(VI) removal under the condition of pH 7 when the initial concentration of Cr(VI) was 5.0 mg/L.

**Figure 10:**
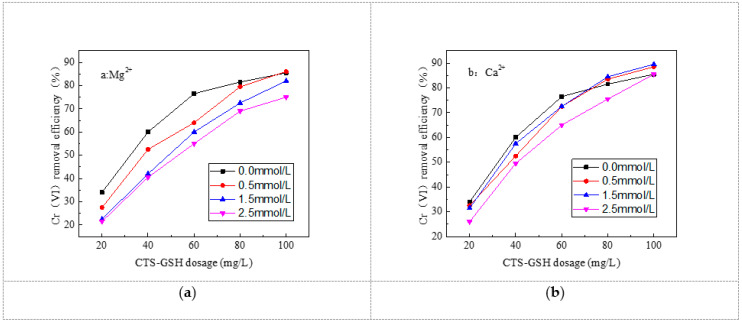
Effect of cations on Cr(VI) removal. (**a**) Effect of Mg2+ on Cr(VI) removal. (**b**) Effect of Ca2+ on Cr(VI) removal.

As can be seen in Figure 10, the removal effect of Cr(VI) was inhibited significantly with the increase in Ca^2+^ and Mg^2+^ concentration. The inhibitory effect was greater at higher concentrations of Ca^2+^ and Mg^2+^. Mg^2+^ had a higher inhibitory effect than Ca^2+^. One can reduce or eliminate the inhibitory effect by increasing the amount of CTS–GSH added. As can be seen in Figure 10a, the Cr(VI) removal rate was 27.5%, 22.5% and 21.5% when 20 mg/L CTS–GSH was added into the water samples with Mg^2+^ concentrations of 0.5, 1.5 and 2.5 mmol/L, respectively. Removal rates of 6.5%, 11.5% and 12.5%, respectively, were achieved in the water sample without Mg^2+^. When 100 mg/L CTS–GSH was added, the Cr(VI) removal rate was 86.0%, 82.0% and 75.0%, respectively. The Cr(VI) removal rate in the water sample with a Mg^2+^ concentration of 0.5 mmol/L increased by 0.5% compared with the water sample without Mg^2+^, and the Cr(VI) removal rate in the water sample with Mg^2+^ concentrations of 1.5 and 2.5 mmol/L decreased by 3.5% and 10.5%, respectively, compared with the water sample without Mg^2+^. As can be seen in Figure 10b, the Cr(VI) removal rate was 34.0%, 32.5%, 31.5% and 26.0% when 20 mg/L CTS–GSH was added into the water samples with Ca^2+^ concentrations of 0.5, 1.5 and 2.5 mmol/L, respectively. In other words, the Cr(VI) removal rate was 1.5%, 2.5% and 8.0% lower than in the water sample without Ca^2+^. When 100 mg/L CTS–GSH was added, the Cr(VI) removal rate was 85.5%, 88.5%, 89.5% and 85.5%, being 3.0%, 4.0% and 0.0% higher, respectively, than the Cr(VI) removal rate in the water sample without Ca^2+^.

The major reason for this phenomenon was that Mg^2+^ and Ca^2+^ coordinated with carboxyl, amino and thiol groups in the CTS–GSH molecules to generate the coordination compound and accordingly reduced the possibility of interaction between CTS–GSH molecules and Cr(VI), thus leading to a decrease in the Cr(VI) removal rate. With the increase in Mg^2+^ and Ca^2+^ concentration, the concentration of positively charged molecules increased in the solution and the cations’ strength also increased. As a result, the coordination reaction between CTS–GSH molecules and Cr(VI) was hindered. Thus, the Cr(VI) removal rate decreased, but this inhibitory effect weakened with the increase in the added amount of CTS–GSH. The inhibitory effect was related to the number of electric charges and the radius of ions. The number of Mg^2+^ charges was similar to the number of Ca^2+^ charges, and Mg^2+^’s radius was smaller than Ca^2+^’s [60]. Thus, Mg^2+^ exhibited a significant inhibitory effect.

#### 3.4.6. Effect of Anions

An experiment was performed to evaluate the effects of anions including SO_4_^2−^ and CO_3_^2−^ on Cr(VI) removal under the condition of pH 7 when the initial concentration of Cr(VI) was 5.0 mg/L.

**Figure 11:**
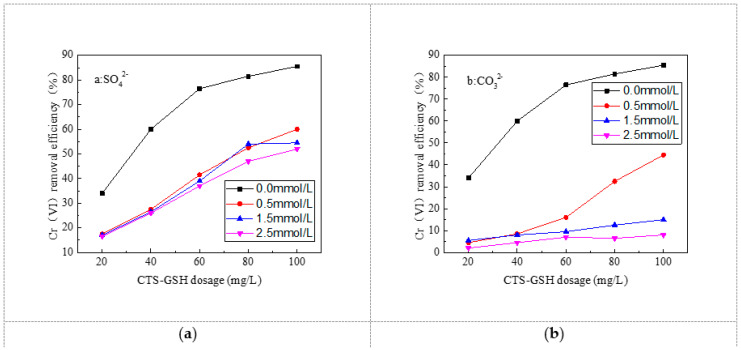
Effect of anions on Cr(VI) removal. (**a**) Effect of SO_4_^2−^ on Cr(VI) removal. (**b**) Effect of CO_3_^2−^ on Cr(VI) removal.

It can be seen from Figure 10 that with the increase in SO_4_^2−^ and CO_3_^2−^ concentration, the Cr(VI) removal rate was inhibited significantly. Additionally, the inhibitory effect was greater when the concentration of SO_4_^2−^ and CO_3_^2−^ was higher in the solution. In addition, CO_3_^2−^ exhibited a greater inhibitory effect on Cr(VI) removal than SO_4_^2−^. As can be seen in Figure 11, when 20 mg/L CTS–GSH was added into the water sample and the concentration of SO_4_^2−^ was 0.5, 1.5 and 2.5 mmol/L, the minimal Cr(VI) removal rate was 17.5%, 17.0% and 16.5%, respectively. Compared with that in the absence of SO_4_^2−^ in the water sample, the Cr(VI) removal rate decreased by 16.5%, 17.0% and 17.5%, respectively. When 100 mg/L CTS–GSH was added, the maximum Cr(VI) removal rate was 60.0%, 54.5% and 52.0%, respectively. Compared with that in the absence of SO_4_^2−^ in the water sample, the Cr(VI) removal rate decreased by 25.5%, 31.0% and 33.5%, respectively. As can be seen in Figure 11b, when 20 mg/L CTS–GSH was added into the water samples when the concentration of CO_3_^2−^ was 0.5, 1.5 and 2.5 mmol/L, the minimum Cr(VI) removal rate was 4.5%, 5.5% and 2.0%, respectively. These findings suggest that the Cr(VI) removal rate was 29.5%, 28.5% and 32.0% lower than in the water samples without CO3^2−^. When 100 mg/L CTS–GSH was added, the maximum Cr(VI) removal rate was 44.5%, 15.0% and 8.0%, being 41.0%, 70.5% and 77.5%, respectively, lower than the Cr(VI) removal rate in the water samples without CO_3_^2−^.

The major reason for this phenomenon was that the coordination reaction might have occurred between Cr(VI) and SO_4_^2−^ or CO_3_^2−^ in the water solution. From the constant coefficient of the coordination compound, the stability of the coordination compounds generated in the coordination reaction between Cr(VI) and SO_4_^2−^ or CO_3_^2−^ was SO_4_^2−^ < CO_3_^2−^ [61]. The higher the concentration of SO_4_^2−^ and CO_3_^2−^ in the water samples, the greater the possibility of the coordination with Cr(VI), resulting in a smaller chance that flocculation of CTS-GSH and Cr(VI) would occur; thus, a decrease in the removal rate occurred. Theoretically, the negative ion might neutralize some positive charges on the molecular chain of CTS–GSH and cause the CTS–GSH molecular chain to spread and increase the possibility of crashing with Cr(VI). In this study, the quantity of SO_4_^2−^ consumed was equal to that of CO_3_^2−^, and the radius of SO_4_^2−^ hydrated ions was smaller than that of CO_3_^2−^ hydrated ions [62], and their location on the CTS–GSH molecular chain was different. Thus, CO_3_^2−^ showed a weaker promotion effect than SO_4_^2−^. Within the range of concentration discussed in this study, the inhibitory effect of SO_4_^2−^ and CO_3_^2−^ took a dominant role. Overall, this study suggested that both SO_4_^2−^ and CO_3_^2−^ exhibit an inhibitory effect on Cr(VI) removal, and the inhibitory effect of CO_3_^2−^ was greater than that of SO_4_^2−^.

## 4. Conclusions

A stable CTS-GSH was synthesized with CTS and GSH through an amide reaction. It had multiple coordination groups that could chelate with Cr(VI) to form metal chelates and eliminate Cr(VI) from an aqueous solution. The FT-IR of CTS-GSH showed that it was a thiol-group-functionalized material, not a simple mixture of CTS and GSH. The SEM of CTS-GSH showed it had a space network structure that was conducive to adsorbing Cr(VI). The parameters affecting the Cr(VI removal rate, such as the initial concentration of Cr(VI), CTS-GSH dosage, pH, slow stirring time and sedimentation time, were examined; the Cr(VI) removal rate increased with increasing CTS-GSH in the system; in the range of pH 5–6, it was conducive to increasing the interaction of CTS-GSH and Cr(VI) ions. The higher the initial Cr(VI) concentration, the more CTS-GSH was needed to achieve a better Cr(VI) removal rate; the highest Cr(VI) removal rate achieved was 99.3% at a slow stirring time of 8 min and sedimentation time of 3 h during the coagulation process under a dosage CTS-GSH of 100 mg/L. The coexistent substances, such as EDAC, SS and TA, either promoted or inhibited the removal of Cr(VI). Common ions, such as Mg^2+^, Ca^2+^, SO_4_^2−^ and CO_3_^2−^, also showed an inhibiting effect, and thus require a greater CTS-GSH dosage for treatment of Cr(VI). Overall, CTS-GSH is an efficient metal chelator. With appropriate control of the above factors, it could reduce the negative impact on Cr(VI) removal, thus obtaining good results for Cr(VI) removal.

## Data Availability

Not applicable.
